# How Can Material Stock Studies Assist the Implementation of the Circular Economy in Cities?

**DOI:** 10.1021/acs.est.2c05275

**Published:** 2022-11-28

**Authors:** Wendy Wuyts, Alessio Miatto, Kronnaphat Khumvongsa, Jing Guo, Pasi Aalto, Lizhen Huang

**Affiliations:** † Department of Manufacturing and Civil Engineering, Norwegian University of Science and Technology, 2815Gjøvik, Norway; ‡ Center for Industrial Ecology, School of the Environment, Yale University, New Haven, Connecticut06511, United States; § Graduate School of Environmental Studies, Nagoya University, Nagoya, Aichi464-8603, Japan; ∥ School of Environment, Tsinghua University, Beijing100084, China; ⊥ Department of Architecture and Technology, Norwegian University of Science and Technology, 7034Trondheim, Norway

**Keywords:** industrial ecology, material stock, circular city, built environment, circular economy

## Abstract

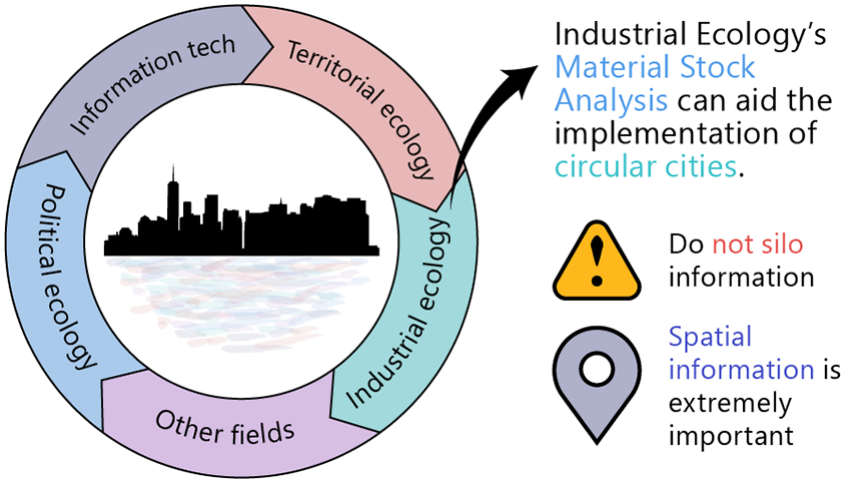

City and regional planners have recently started exploring a circular approach to urban development. Meanwhile, industrial ecologists have been designing and refining methodologies to quantify and locate material flows and stocks within systems. This Perspective explores to which extent material stock studies can contribute to urban circularity, focusing on the built environment. We conducted a critical literature review of material stock studies that claim they contribute to circular cities. We classified each article according to a matrix we developed leveraging existing circular built environment frameworks of urban planning, architecture, and civil engineering and included the terminology of material stock studies. We found that, out of 271 studies, only 132 provided information that could be relevant to the implementation of circular cities, albeit to vastly different degrees of effectiveness. Of these 132, only 26 reported their results in a spatially explicit manner, which is fundamental to the effective actuation of circular city strategies. We argue that future research should strive to provide spatial data, avoid being siloed, and increase engagement with other sociopolitical fields to address the different needs of the relevant stakeholders for urban circularity.

## Abstract 2

Material stock studies, despite their popularity, offer little information to circular city planners. Here, we look at what material stock studies can do to aid the creation of circular cities.

## 1 Introduction

The traditional linear economy, where materials are extracted, used, and discarded, is increasingly challenged by the circular economy (CE).^1,2^ Despite lacking a formal definition,^3^ the CE is an umbrella of principles aimed at reducing the environmental impacts deriving from traditional economic practices while maximizing the potential (re)use of materials. Although some CE concepts celebrated their 50th anniversary,^1^ different stakeholders have only widely started to embrace CE practices in the past decade. CE business models and strategies emphasize solutions for specific sectors but have the tendency to disregard the complexities of place-specific systems.^4^ Businesses and industries do not exist in a vacuum but are interlinked into complex infrastructure systems, markets, and regulations. Therefore, spatial contextualization is often needed, especially at the onset of planning and implementation processes.^5^

Urban areas can be studied from multiple perspectives: people, food, energy, water, infrastructure, and more. While all these aspects are interlinked,^6,7^ this Perspective focuses solely on buildings and infrastructure in urban areas. Cities, which now host more than half of the global population, are seen as the locus of many environmental issues yet also the place for innovation.^8^ The growth of cities is evident from historical material consumption accounts (e.g., refs (9 and 10)). Urban growth also gathers the interest of urban planners, policymakers, and scholars, whose research started exploring a circular approach to urban development.^4,11−13^

Material stocks refer to goods used for an extensive period, typically more than one year.^14^ Because material stocks are, by definition, durable goods, buildings and infrastructure constitute the near entirety of global material stocks.^15^ Interest in the role of material stocks has been surging in recent years, as these materials are considered the enablers of human development and economic activities.^16^ Recent years have seen the publication of a few relevant reviews on this topic. Lanau et al.^17^ published an overview of material stock studies that targets a technical audience. Fu et al.^18^ described how existing material stock studies could inform CE strategies based on data availability and quality. Concurrently, to popularize the circular city theory, industrial ecologists have been designing and refining methodologies to quantify and locate material flows and stocks within systems.^19^ It is implicitly assumed, and often briefly mentioned in the discussion sections of scientific articles, that the quantitative results of material flow and stock studies can assist policymakers and planners in making decisions that can increase city circularity (e.g., ref (5)). However, it is unclear to what extent, for which practices, and what results are best suited to provide this information.

In this article, rather than trying ex-post to find a use of material stock and flow results in circular city theories, we explore which information is needed to enable circular cities and how material stock studies can support it. We identify the contributions and gaps of existing material stock studies relevant to urban circular practices and discuss how future research could fill those gaps.

## 2 Theory

The development of circular cities requires an interdisciplinary vision that depends on the cooperation of different stakeholders across different scales. In this section, we explore existing theories on circular city practices.

### 2.1 Two Main Approaches to Circular Cities

There are two main approaches to circular cities. The first and most common approach is to make urban economies as circular as possible and thus see circular cities as a sum of circular businesses.^20^ This approach disregards contextual factors like infrastructure capacity, human resources, or the interaction with neighboring places. The second approach is to (re)contextualize the industries, i.e., considering local assets and interactions with other places they depend on (e.g., neighboring regions). This second approach is getting vouched for by various researchers (e.g., ref (21)). However, the main drawback of this method is its far more complex implementation^22^ and monitoring,^23^ as many data are often only available at the national level. Scholars of the built environment often take a deterritorialized approach, neglecting the complexities of urban systems^4,12^ and ignoring nexuses with energy and water, although these interrelations have already been remarked.^6,7,24^ One of the most promising ways to contextualize CE strategies is combining industrial, territorial, urban political, and Marxist ecology tools.^12^ This combination aligns well with the local contextualization approach as this is the only way to enable a socially just CE for a broad arena of citizens. These tools, and the studies they stem from, contribute to the concept of urban metabolism.^25^ However, the insights of these different subfields are often siloed and rarely combined.^26^

Marin and De Meulder^12^ explain how the two approaches to circular cities have both benefits and barriers. The authors indicate that a holistic approach to circular cities can find the root causes of many social and environmental problems, but such an approach requires the expertise of various theories. Unsurprisingly, only a few scholars illustrated the advantages of combining industrial ecology methods, like material stock studies with qualitative research informed by political ecology or stakeholder analysis (e.g., refs (27 and 28)).

### 2.2 Strategies at the Macrolevel

Three CE strategies where material stock studies can contribute to circular cities have emerged in the literature. The first strategy is exploiting the already available local resources. Existing local resources, often termed “in-use stocks”,^29^ can be harvested, recycled, or reused, offsetting primary material extraction. This practice is widely labeled under the term “urban mining”.^30^ Urban mining can apply to materials, buildings, and areas, the so-called “wastescapes”.^27,31,32^ Material stock studies can contribute to urban mining strategies by providing spatially explicit information to locate and quantify salvageable materials.^33−35^

A second strategy is regenerating urban areas. Regeneration does not have an exact definition. It can mean removing unutilized material stock that meaninglessly occupies land so there will be more green spaces for health services^36^ or more wetlands and other spaces that can help in climate, flood, and other crises. It can also mean converting existing buildings to other uses and ecosystem services.^37^

A third strategy is using sustainable materials and design strategies to minimize environmental impacts. Some researchers are interested in assessing the existing stock quality and replacing high-impact construction materials (e.g., concrete, steel) with low-impact ones (e.g., bamboo, timber) by conducting life cycle assessments of the whole building lifecycle.^38^ Others seek the best design strategies to minimize energy consumption.^39^

These three strategies can significantly impact increasing the circularity and overall sustainability of the urban environment. However, they do not reflect on the local specificities as they apply, to an extent, to any urban area of the world.

### 2.3 Strategies at the Microlevel

At the microlevel, various scholars often proposed the so-called Rs strategies, e.g., reuse, reduce, recycle.^40−42^ As our focus is on the built environment, especially the building elements, we focus first on strategies interesting for the building industry:^43^ (1) onsite reuse; (2) repairing; (3) offsite component reuse; (4) reprocess/remanufacture/recycle. The benefits and limits of these four practices are discussed in detail in the articles of Fivet and Brütting^43^ and Cai and Waldmann.^44^ Another essential strategy is lifetime extension.^45−47^ Buildings that live longer result in lower primary material extraction and waste generation and tend to benefit from retrofitting and refurbishment more than from complete demolitions and reconstructions. Verga and Khan^48^ provide many practical examples of these theoretical strategies.

## 3 Method

This study is based on a critical literature review queried through Scopus. The query we used is displayed in eq 1:
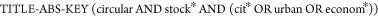
1where TITLE-ABS-KEY looks into article titles, abstracts, and keywords. The terms we search for are circular stock city, circular stock urban, or circular stock economy. The asterisk at the end of the works considers that some of these terms can be used plurally. We deliberately included the word stock as we seek material stock studies (omitting it would have returned hundreds of articles that deal with circular cities but do not involve material stock analysis).

We limited our search to articles that are peer-reviewed and written in English. The query returned 262 articles, which we classified according to a matrix we developed leveraging existing circular built environment frameworks of urban planning, architecture, and civil engineering.^4,20^ Further, we adapted the matrix to include the terminology of material stock studies, as proposed by Lanau et al.^17^

The matrix consists of geographical and spatial categories, accounted materials, end-use-sectors, use-state (i.e., in-use, abandoned), period of analysis, modeling method, environmental impacts (e.g., waste, pollution), and different CE applications (e.g., reuse, regeneration, renovation). The matrix classification helped us identify the knowledge gaps and needs that secondary material suppliers and clients require to increase the circularity of the construction sector. The matrix is available for download in the Supporting Information. The discussion is informed by academic literature but also by our experiences and participatory and marginal observations as architects, engineers, designers, researchers, and/or innovation actors in the circular built environment.

## 4 Results

**Figure 1:**
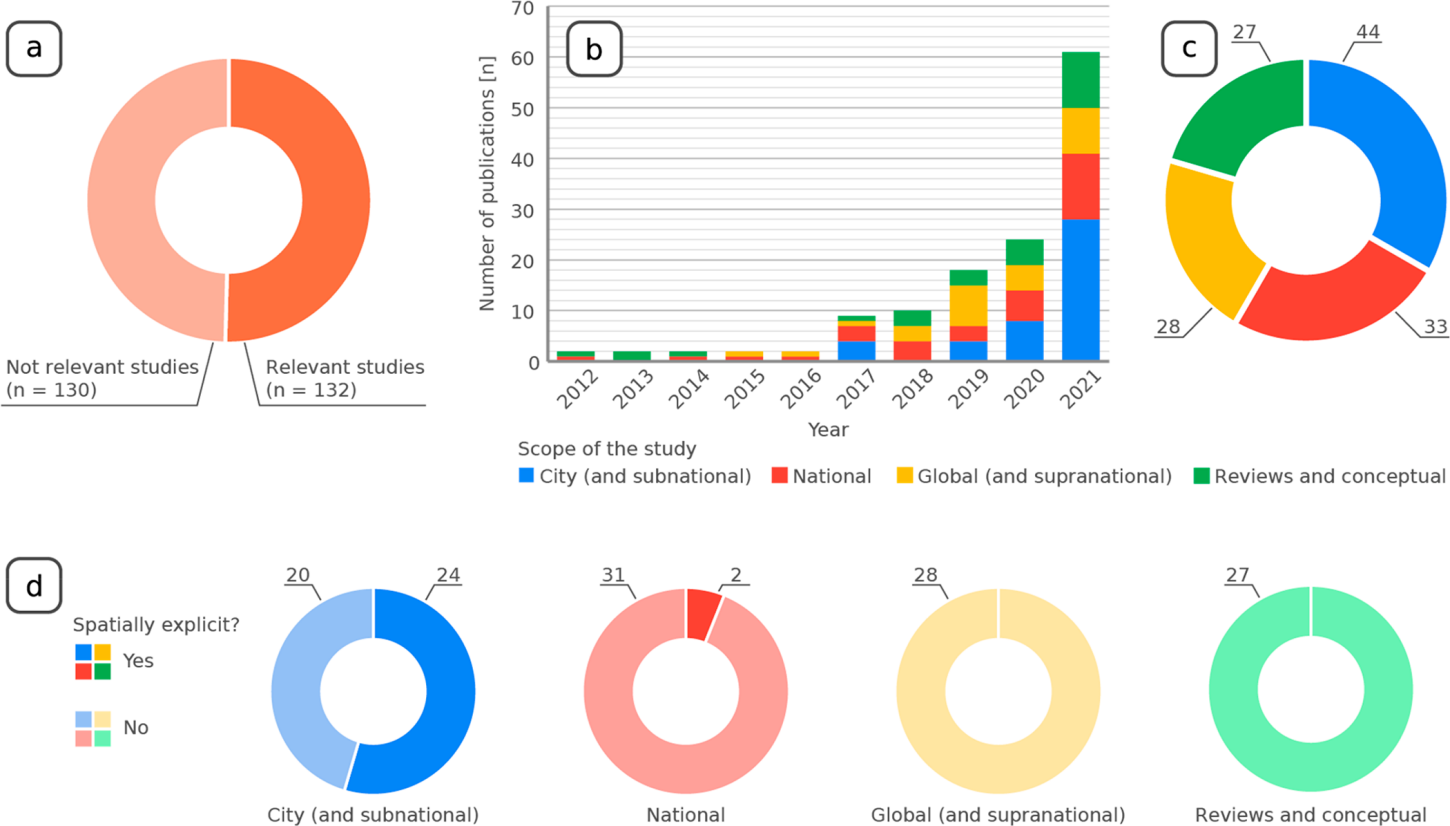
(a) Share of publications relevant to this Perspective. (b) Number of publications from 2012 to 2021 divided by their geographical scope. (c) Share of publications divided by their geographical scope. (d) Share of publications that provide spatially explicit data divided by their geographical scope.

Of the 262 articles we identified, 130 were irrelevant to our research because they did not include construction stock data or mentioned circular cities only en passant (Figure 1a). Recent years have seen a surge in publications of material stock studies (Figure 1b). In 2021 alone, 61 material stock studies were published, 46% of all the studies relevant to this research. The 132 relevant articles were divided into empirical urban stock studies (44 articles), national material stock studies (33 articles), multinational, continental, or global studies (28 articles), and conceptual and review papers (27 articles) (Figure 1c). As the goal of this Perspective lies in understanding the contribution of material stock studies to the design of circularity in cities, we investigated the presence of spatial information. Figure 1d illustrates how only 24 of the 44 urban studies were spatially explicit, and only 2 of the 61 national or global studies provided any form of spatial data. Almost all the spatially explicit studies (19 of 26) were bottom-up studies (i.e., studies that use an inventory of building areas and material intensities). Only 10 papers provided the explicit location of local urban mining areas but no time frame in which these materials would become available.

We identified three types of studies that are beneficial for circular cities. The first type is material stock studies that help monitor and trace materials and waste within cities (e.g., ref (49)). Some of these studies include environmental impacts (e.g., ref (10)). These studies highlight how material stock analyses can aid the achievement of set goals (e.g., carbon neutrality of the building sector). The second type is critiquing papers: articles that do not necessarily provide solutions but pinpoint blind spots and criticalities within cities (e.g., ref (50)). For example, several studies indicate the proximity to treatment plants and markets as a key factor for implementing circular practices.^50^ Other studies challenge the customary focus on the city administrative boundaries for analysis and data collection (e.g., refs (51 and 52)). The third type refers to studies examining policies’ effects on material stock accumulation (e.g., ref (53)). These studies inform policymakers about which policies might be detrimental to CE effectiveness, like increased domestic material consumption and emissions.

Several papers did not put material stock data as their primary focus but combined them with other results. These articles are often associated with the field of political-industrial ecology.^26^ Some interesting papers came from design studies (e.g., refs (54 and 55)), where material stock analysis is only a fraction of the whole research goal. Other articles criticized the current circularity strategies in cities. For example, Van den Berghe and Verhagen^50^ combined findings of a material stock study^56^ with an origin-destination analysis to calculate emissions for secondary materials transports. This study raises interesting questions about the external costs and limitations of the logistics of materials between different lifecycle phases, not only for the traditional linear economy but also for circular economy practices.

Most papers accounted for structural materials, chiefly timber, concrete, and steel. Structural steel and timber are particularly interesting to structural engineers because they can be simply reused, at least in some instances (see ref (43)). Except for timber, renewable materials such as rammed-earth or bamboo are nearly absent from our sample of material stock studies. Some scholars argue that material stock studies should be done at the level of building components because of prospective market values,^57^ yet only a few cases looked into this perspective (e.g., refs (58 and 59)). Arguably, there should be a clear link between the study of material compositions and the current and future availability of cost-effective technologies for recycling and pretreatment for reuse.^60^

Our matrix checked for the presence of other strategies, such as substitution and regeneration, but none of our sampled studies contributed empirical evidence to these major strategies. Further, we highlight that only one article from Stephan and Athanassiadis^61^ has explicitly analyzed the nexus between construction materials and other resources, such as food or water, in urban areas. This somewhat disappointing finding confirms the need for better interdisciplinary research and communication among experts in different fields.

## 5 Discussions

### 5.1 Limitations of Material Stock Studies from the Circular Cities’ Perspective

#### 5.1.1 Limitations of Top-Down Material Stock Analyses

Many material stock studies employ a top-down approach (i.e., a compilation of material stocks using macroeconomic data and lifetime assumptions). Top-down stock data can offer insights for drafting national policies and monitoring environmental performances. Nonetheless, these top-down analyses are often conducted at the national level^62^. Additionally, prospective top-down studies are often based on assumptions (e.g., service time, population growth) that have limited usefulness to planners because the studies do not give precise recommendations that are actionable in urban planning processes. Importantly, because the built environment is immovable, unlike other material stocks such as cars or cellphones, only spatially explicit material stock studies are relevant to promoting circular city strategies.

#### 5.1.2 Limitations of Material Stock Studies That Focus Only on One Aspect

Material stock studies are often conducted on a single aspect, be it materials, building components, or entire buildings. Moreover, most studies focus on a single scale (e.g., national, regional, urban). Material stock studies should cover various scales to be genuinely effective, and research should investigate how the different scales interlink. Huuhka and Kolkwitz^51^ proposed nested hierarchies of different scales using a bottom-up analysis for existing buildings in Tampere. Busch et al.^63^ built a hierarchical nested representation of material stocks on both materials and commodities to assess material criticality.

One emerging question in circular city studies is whether it makes sense to limit study scopes to city boundaries, which tend to be arbitrary and, at times, fail to offer a holistic view of the urban area. Marin and De Meulder^12^ argue the need to examine the broader ecosystem in which cities are embedded. While it would be theoretically possible to design an ideal circular city in a vacuum, we cannot ignore that, in reality, cities are never fully self-sufficient and isolated. In the field of geography, the classic separation between urban and rural areas has already been criticized for decades.^64^ To design truly circular cities, we ought to extend our perspective to include not only the city itself but also its surrounding areas.^65^

Ultimately, the choice to focus solely on materials, components, entire buildings, or multiple aspects depends on the specific applications that need to be addressed. If an ex ante choice had to be made, it is our position that building components offer more actionable information to practitioners. Research such as that conducted by Arora and colleagues,^59^ where they quantified the annual building components in Singapore, showcases how this information can be used for planning reuse surveys and finding a market for secondary building components.

#### 5.1.3 Lack of Information to Support Urban Mining of (Obsolete) Material Stocks

The circular city strategy to which material stock studies can contribute strongly is urban mining. However, only a few studies provide details on obsolete building stocks that can be harvested (e.g., ref (27)). For the most part, researchers report urban stocks without clearly differentiating between what is in use and what is abandoned. Thus, not much information can be gathered on the reusability potential of these stocks. Urban mining and reuse have technical requirements, specific economic structures, and ad hoc policies.^44^ To further complicate things, standards and design choices further limit the reusability of materials.^66^ The successful implementation of urban mining depends on the availability of data related to material quality, quantity, location, temporal availability, and accessibility.^34^

Nevertheless, there is often a lack of information concerning the presence of contaminants that can limit reuse. In other words, material stock studies can deliver extensive data about material availability, but as Winterstetter et al. highlighted,^34^ not about recoverability and reuse potential. Most studies acknowledge that they do not provide enough details about material composition and contamination for recycling, remanufacturing, and reuse purposes (e.g., ref (67)).

### 5.2 The Usefulness of Material Stock Studies to Circular Cities

#### 5.2.1 Material Stock Studies Can Inform Material Exchange Platforms

Material stock studies are capable, at least in theory, of identifying priority areas for urban mining. To identify these areas, material stock studies must first recognize what areas are expected to experience an uptick in demolition activities. This macro- and mesolevel information is then followed by a collection of surveys to detail existing building stocks and feed material exchange platforms (microlevel information). After considering technological limits and economic feasibility for microlevel data, material stock studies may expand their scope to include the location of potential clients for secondary building materials and components. We call attention to the fact that such considerations are rarely found in most articles we analyzed (one exception is, for example, Lanau and Liu^68^). In most cases, material stock studies quantify the existing stocks, and sometimes their related inflows and outflows, without including any urban mining information.

The general lack of spatial information frustrates the effectiveness of circular city strategies, as it is impossible to draw any conclusive facts on the local availability of materials or generate techno-economic analyses for different decisions. The successful creation of secondary construction materials markets calls cities to look beyond their boundaries and invest in creating both physical and digital material repositories to enable trade in national or global markets.

Moreover, we did not find any study that empirically demonstrated how material stock studies helped microlevel stakeholders. The main barriers are the lack of a solid database solution on availability (i.e., one-stop platform of reclaimed building materials), accessibility (i.e., open access if applicable), interoperability (i.e., secure and efficient data exchange), and user-friendly interfaces for a vast array of users. The different actors involved in circular cities do not have an equal set of skills, information, users, and collaboration requirements.^69^ Hence, we highlight the need for transdisciplinary and multiscalar frameworks to enable information sharing.^70^ Developing these frameworks requires understanding information requirements for different actors (planners, architects, sustainability researchers), data requirements and management, and a fair distribution of costs and benefits. An apt example is the circWOOD project in the Norwegian SirkTre consortium.^71^ This project aims to bring spatially explicit material stock data to a digital platform/twin that renders information on the location, quantity, and quality of potential waste timber for companies interested in reusing this reclaimed timber in construction. SirkTre project partners are currently locating the most suitable locations for storage and pretreatment hubs and evaluating any supporting infrastructure that might be needed. The experience derived from this project could be used for future material exchange endeavors and could facilitate the actualization of circular initiatives for construction.

#### 5.2.2 Material Stock Studies Can Contribute to Locating Future Circular Hubs

In circular city projects, various businesses and industries aspiring to spearhead the CE in their city/region/country are simultaneously at play. However, they often cannot coordinate without intermediaries. These intermediaries are material banks, logistics hubs, or circular hubs. They are a significant part of a product value chain and depend heavily on their surroundings. Their location, client access, and resource availability determine economic, environmental, and social costs. Material stock studies can contribute to implementing circular cities by mapping the location and tallying the amount of available resource stocks. Importantly, these material stock studies must be maintained up to date if their relevance and usefulness are to be preserved.

Architectural firms, which are especially environmentally conscious, with examples in Belgium,^72^ Norway,^73^ and Switzerland,^74^ have used the results of material stock studies to help with their design process. Material stock data on available resources and infrastructure are integrated with historical assessments of the local context and stakeholder meetings to design desirable buildings that are sustainable and make use of local materials. The architects’ attitude toward material stock data indicates the importance of having opportune infrastructure (e.g., warehouses, circular hubs) to render the acquisition of secondary materials feasible and easy or, at the very least, easier than going from building owner to building owner to try to purchase secondary materials. Considered the importance of material exchange infrastructure, we remark on the importance of material stock studies to include the presence of this infrastructure in their sustainability assessments.

### 5.3 Who Benefits the Most from Material Stock Studies?

Despite many boilerplate remarks about the contribution to the circularity of the construction sector, almost no article in our sample could explicitly indicate who should benefit from the study results in an actionable way. A handful of articles suggested which stakeholders could benefit from their results, especially when combined with contextual insights (e.g., ref (27)). One important note is that most material stock studies offer strategic insights only to macrolevel users (e.g., national policymakers), but they are rarely used to support local strategic planning.^75^ To bring material stock studies to the next level, we encourage the inclusion of methods from the fields of political ecology, economic geography, or other social sciences to understand the local contexts and identify strategic leverage points that lead to a fair distribution of services and resources.

To effectively implement a circular built environment, we call for innovative studies that combine material stock analysis of different scopes (i.e., materials, components) with consideration of local characteristics (e.g., transportation requirements to recycling facilities). Material stock information should be further developed to support decision-making at the building project level, for example, by developing a BIM object library for reusable building elements or creating the user-friendly digital twin for deconstruction. Further, prospective studies that consider the existence of vacant and abandoned buildings will support the creation of a dynamic material repository that could be potentially harvested for reuse. Future research should create microlevel material stock data repositories and integrate building information modeling to facilitate the sourcing of secondary building materials and components.^30,49^ However, who should manage and maintain these data sets and how digital material exchange platforms will be financially sustainable remains to be seen.

As Marin and De Meulder noted,^12^ no discipline alone is sufficient to contribute to informed and well-rounded decision-making. Material stock research makes no exception, and analyses from other fields should complement it. Moreover, more research should be provided in a spatially explicit fashion rather than in a nationally aggregated form. Reliable, available, and timely material stock data is a necessary but not sufficient condition for the effective implementation of circular cities. Only a clear understanding of the context in which these cities are built, which stems from fields like geography and landscape architecture, and transdisciplinary methods will enable the tightening, or closing, of material loops in constructions.

## Supporting Information Available

Database and analysis of studies (XLSX)
